# 3D Printed Lenses for Vertical Beam Collimation of Optical Phased Arrays

**DOI:** 10.1089/3dp.2022.0314

**Published:** 2024-06-18

**Authors:** Sidra Tul Muntaha, Ari Hokkanen, Mikko Harjanne, Matteo Cherchi, Pekka Suopajärvi, Petri Karvinen, Markku Pekkarinen, Matthieu Roussey, Timo Aalto

**Affiliations:** ^1^ VTT Technical Research Centre of Finland, Espoo, Finland.; ^2^ Department of Physics and Mathematics, University of Eastern Finland, Joensuu, Finland.

**Keywords:** optical phased arrays, 3D printed lens, LiDAR, collimation, silicon photonics

## Abstract

This article presents the design, fabrication, and characterization of edge-coupled 1D optical phased arrays (OPAs) combined with collimating lenses. Our concept was tested with two OPAs having different collimation ranges. Both OPA designs have 3-μm waveguide spacing and the maximum beam steering range is about 30° based on wavelength tuning around 1550 nm. The first generation had 37 channels with 108 μm of waveguide array width and the second generation had 512 channels with 1.5 mm array width. As the array outputs are edge coupled, suitable lenses are required to collimate the beam vertically. We report the comparison between a commercially available straight cylindrical lens and custom 3D printed curved cylindrical lenses. In the experiments, we demonstrate 1D beam steering of the light outcoupled from the waveguide facets and collimated by these lenses and analyzed parameters such as Rayleigh range and beam divergence. These parameters are estimated to be 9.9 mm and 7.0 mrad (0.4°), respectively, for the commercial lens, whereas 40.1 mm and 3.5 mrad (0.2°) for the dedicated 3D printed lens, showing a clear improvement.

## Introduction

Light detection and ranging (LiDAR) control systems have a variety of applications, for example, terrain mapping by identifying road painting along with the lanes^1^ and pedestrian recognition systems for autonomous vehicles.^2^ In fact, LiDAR systems have been identified as an important solution for autonomous vehicles, as it helps to improve their reliability and efficiency. LiDAR sensors and building blocks for their integration on silicon photonics platforms have been widely reported.^3–5^

A significant part of a LiDAR system is the beam steering mechanism. Apart from LiDAR applications, beam steering can be employed by a variety of other applications such as beam forming, laser engraving,^6^ microscopes,^7^ and optical wireless communications. There are mainly two types of beam steering systems, that is, mechanical and nonmechanical solutions. The mechanical solutions include rotating prisms,^8^ micro-electromechanical systems,^9^ galvo scanners,^10^ rotors and oscillating mirrors,^11^ and so on.

These mechanical solutions are quite precise and widely used, but they are prone to failure,^12^ because of the moving components. The nonmechanical solutions include electro-optic and acousto-optic deflectors,^12^ liquid crystal spatial light modulators,^13^ and beam steering based on phased arrays.^14^ Through these nonmechanical solutions to the beam steering, the device size could be reduced to make it more cost-effective, reliable, immune to vibration, and compact. The speed can be enhanced, and power consumption can be reduced.^15^ These characteristics are important and attractive, while considering the LiDAR applications and autonomous vehicles.

Among these nonmechanical solutions, optical phased arrays (OPAs) have gained a significant popularity, allowing very precise and stable beam steering. On-chip OPAs are in much lime in recent years.^16–19^

End-fire OPAs allow only 1D beam steering^20–22^ where the power is radiated at the end facets of the waveguide in contrast with waveguide grating antenna OPAs, which enable 2D beam steering^23–25^ along horizontal and vertical direction.

The goal in this research work is to demonstrate a wavelength tuning-based 1D beam steering based on the on-chip OPAs and 3D printed curved cylindrical lenses on 3-μm silicon-on-insulator (SOI) platform. The output from the OPA chips has higher beam divergence in vertical direction; thus, there is a need for off-chip curved cylindrical lenses to collimate vertically the beam.

## Development of OPAs on Micron-Scale Silicon Photonics

OPAs offer a technique of beam shaping and steering with wavelength tuning without any mechanical part. The output of an OPA consists of an array of waveguides, each acting as a radiating element. The waveguides are usually spaced uniformly. Thus, by introducing a uniform optical phase shift Δ*φ* between adjacent waveguides, the phase-front of the output beam is tilted, and the direction of the output beam is changed. The steering angle *θ* can be calculated from Equation (1)

(1) $$\theta =\sin^{-1}\left(\frac{\Delta \varphi \lambda }{2\pi d}\right),$$

where *λ* is the wavelength and *d* is the waveguide pitch.^26^

In the case of wavelength tunable OPAs, the phase shift is caused by a difference in the optical path lengths in each of the output waveguides of the array. A path length difference of Δ*L* between adjacent output waveguides causes a phase difference, which is presented in Equation (2)^26^

(2) $$\Delta \varphi =\frac{2\pi }{\lambda }n_{eff}\Delta L,$$

where *n*_eff_ is the effective index of the waveguide. To cover the whole steering range, the phase needs to be swept over the full range $\Delta \varphi =\left[0\ldots 2\pi \right]$. The wavelength tuning range Δ*λ* required for this is given in Equation (3)^26^

(3) $$\Delta \lambda =\frac{\lambda^{2}}{\Delta Ln_{g}},$$

where *n*_g_ is the group index of the waveguide.

The first generation of our OPAs consisted of 37 output channels with 3-μm waveguide spacing. The count and the spacing were determined by the available layout footprint and the minimum recommended linewidth in the fabrication process, respectively. The OPA was designed with Δ*L* = 8.25 μm, which corresponds to a Δ*λ* of 85 nm centered at 1550 nm.

**FIG. 1.**
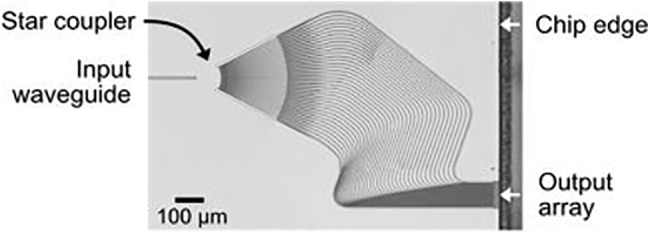
Microscope image of wavelength tuning 37-channel OPA fabricated on a 3-μm-thick silicon platform. OPA, optical phased array.

The total width of the output array is around 108 μm and this kind of array is suitable for collimation at short distance. The microscope image presented in Figure 1 is the top view of the chip (slightly tilted).

The design is based on a star coupler, which consists of an input waveguide coupled to a free-propagation region (FPR). The light beam expands horizontally during its propagation along the FPR. During expansion, the light can be approximated as a Gaussian beam with waist localized at the end of the input waveguide. The amount of light coupled to the output waveguides also follows a Gaussian distribution, with more light coupled to the central waveguides. This distribution suppresses the side lobes of the LiDAR output beam. The design is based on a single beam that could be steered with the wavelength tuning.

The OPA was fabricated on 3-μm SOI in a multiproject wafer run and the beam steering performance characterized using an external C-band tunable laser. All the parameters are summarized in Table 1. The horizontal beam steering angle is measured to be *θ* = 30°, which translates into a wavelength range Δ*λ* = 85 nm. The wavelength steering sensitivity is about 0.35°/nm. The total vertical beam expansion is about 50°. The relatively narrow 108-μm-wide 37 waveguide array limits the horizontal collimation distance to 10–20 cm with the help of collimating lenses.

**Table 1.** Summary of the Parameters of the Two Generation of Optical Phased Array Used in This Work

OPA generation	1	2
Out waveguides	37	512
Δ*λ*	85 nm	85 nm
Steering sensitivity	0.35°/nm	0.6°/nm

The central operation wavelength is 1550 nm.

The second generation of OPA contains 512 waveguides and the output is wider than 1 mm waveguide array to increase the horizontal collimation distance. The waveguide pitch is again 3 μm. The input waveguide feeds a 1 × 64 star coupler, after which each channel has 1 to 8 splitting using a binary tree of 1 × 2 multimode interference splitters in three stages. The maximum beam steering angle is about the same, that is, *θ* = 30° and the wavelength steering sensitivity is 0.6°/nm. The second generation of OPA increased the lateral collimation distance.

**FIG. 2.**
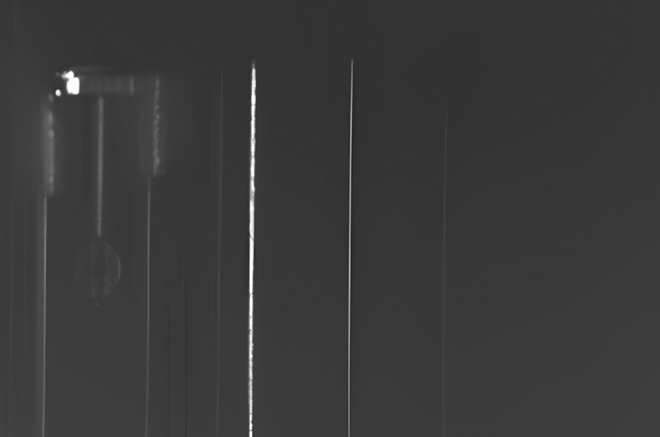
Output of the 512-channel OPA scanned 48 cm away from chip.

However, phase error contributed toward having the spurious peaks apart from the central peaks as it can be seen in the Figure 2. This picture shows the output light of the 512-chanel OPA imaged with a near-infrared camera. The bright (main) line corresponds to the central peak at the wavelength 1528 nm, which gives a straight output beam. Dim lines are representing the spurious side peaks mainly due to a variation in the effective indices before and after the splitter. Over the entire device, the main steering lines have 50 nm spacing with wavelength tuning and 7 dim lines appear between them.

Based on the analysis of problems with the second generation of OPA, a third generation is currently in the design stage, which excludes the splitters and is based on a wide waveguide array.

## Design and Fabrication of 3D Printed Collimating Lens

Free-form optics helps to avoid the mechanical complexity of systems by reducing the number of required optical surfaces to achieve the desired function. However, manufacturing of this class of optics is still complicated and may be time-consuming. The choice of method depends upon the device design, because this is mostly application specific.

Our chips provide high beam divergence in the vertical direction, which therefore requires vertical collimation. When the propagation distance from the chip increases, it is important to consider covering the whole scanning range expected during beam steering. The commercially available straight cylindrical lenses can provide beam collimation for just small beam steering angles (0 < *θ* < 30°). Straight beam shows optical performance between different lens materials and straight cylindrical lenses are good enough for that. However, the costs for machined curved cylindrical lenses are very high and many design generations would multiply this high cost. We did two lens design rounds with 3D printed lenses and some printing process variations. The same kind of work would be needed with commercial lenses.

**FIG. 3.**
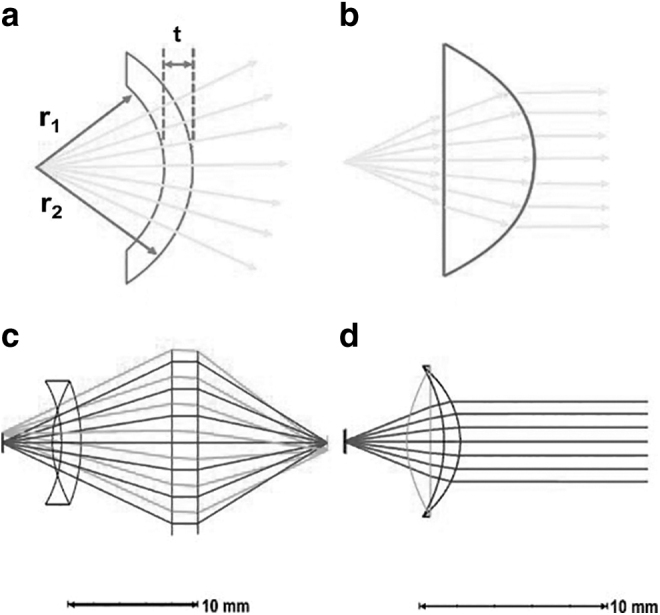
Principle of the collimation lens: **(a)** horizontal propagation of beam (beam steering over a large wavelength range), **(b)** vertical propagation of beam (collimation), **(c)** horizontal paraxial surface for lens optimization, and **(d)** vertical paraxial surface.

As most of the applications of LiDARs request large beam steering angles, it yields a need for customized free form optics element, that is, dedicated curved cylindrical lenses. We propose a curved 3D printed lens enabling maintenance of the beam shape at larger angles horizontally and simultaneously provide a collimation of the divergent beam vertically. This is easily achieved by placing the source at the horizontal center of curvature of a meniscus lens, so that the lens has no effect on the light beam in horizontal direction. In the vertical direction, the shape of the lens is optimized to collimate the beam as shown in Figure 3a and b. It leads to a complex 3D shape for the lens since the curvature is different on both sides and both directions. 3D printing offers an inexpensive method to free form lens manufacturing.

In optical design software, Zemax, the lens is made using *bi-conic* and *toroidal* surfaces as shown in Figure 3c and d. The optimization of the lens was performed using standard merit function and two extra paraxial surfaces; the first one to collimate the horizontal direction and the other one to focus both directions.

The Printoptical^®^ technology is used for 3D printing of the lens, taking the freeform optics in consideration. A detailed concept for 3D printing process is discussed by Assefa et al.^27^ The process is based on industrial inkjet printing; three industrial quality printheads offer three thousand nozzles for use in parallel. Each nozzle can deposit a few picoliter0sized droplet of ultraviolet (UV) curable resin on the substrate. Adjacent droplets merge into a smooth layer, which is afterward cured applying low-intensity UV light, that is, UV pinning. After the layer-by-layer printing process, the final UV curing is done using a mercury vapor lamp. The printing area dimensions are 60 × 60 mm and the print speed in the vertical direction is 5 mm/h.

In this application, the designed lens size is relatively large (136 mm^3^). Two-photon polymerization (2PP)^28^ is a good way to manufacture 3D optical nanostructures and microstructures when the object's volume is in a few mm^3^ scale. Although the possibility of stitching is there, it would be too time-consuming to be feasible. Recently, the two-photon grayscale lithography 3D printing has been introduced, which has improved surface quality and about five times faster manufacturing speed compared to normal 2PP printing process.^29^

Other alternative 3D printing methods for creating the relatively large optical lens with double-sided shapes would require postprocessing. We suggest that a potential method would be using projection micro-stereolithography (PμSL), high-resolution StereoLithography Apparatus (SLA) or lower resolution 2PP^30^ for 3D printing, and applying a spin coating for both sides of the lens would improve surface roughness for acceptable range.^31^ On other hand, using the inkjet process, it is possible to scale up the system and manufacture tens or even hundreds of lenses per an hour if small patch manufacturing is needed.

In this work, two prototypes were manufactured using two different resins: LUX opticlear whose refractive index *n* = 1.52 at *λ* = 1550 nm and micro resist technology mr-UVCur26SF resin whose refractive index *n* = 1.51 at *λ* = 1550 nm.

**FIG. 4.**
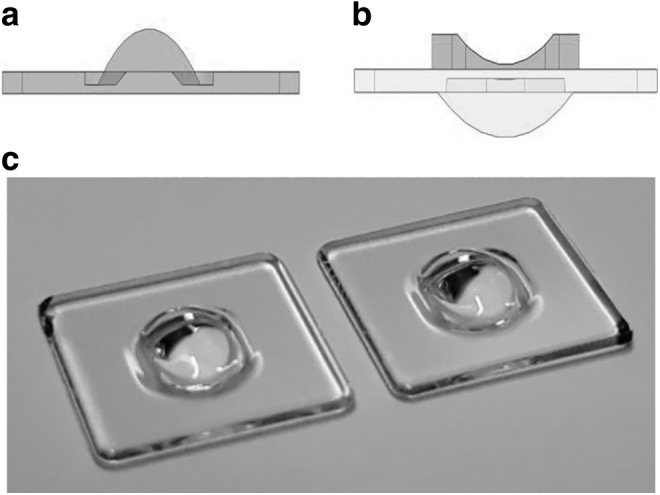
Printing the lens in two phases: **(a)** bi-conic lens, **(b)** the first print is flipped and the second side is printed, and **(c)** 3D printed curved cylindrical lens.

Initial step for the manufacturing of the lens is based on transferring the design into a printable CAD format. Then, the model is sliced into two-dimensional thin layers to be printed. To ensure printability of the lens, the central thickness was adjusted to allow the lens to be printed in two phases: first, the top side, including biconic surface, and after the lens has been turned around, the bottom side with toroidal surface, as shown in Figure 4. In the first phase, tiny alignment marks are printed near each corner of the flat plane apart from the lens, which allows a correct placement with the 3D printer camera-based alignment system for the second phase of printing.

A glass substrate is used in the first phase in which the surface is pretreated for 15 min in a mixture of trimethylhydroxysilane and HFE-7100. It allows the proper attachment of the droplet on the glass substrate. Later, rinsing is done in HFE-7100 for 15 min.^32^ In the second phase, the lens is mounted on an acrylic holder. The speed of the printing process was 2.5 mm/h in height, so the printing time of a single lens of this design was less than one hour. In case of small series production, the whole print area (7 × 7 cm) could have been filled with lenses and printed at the same time.

## Beam Steering Experiments

This section presents a detailed discussion about the results obtained from the first-generation OPA chip (37 channels), either with the commercial lens or without it. An analysis based on 3D printed lenses is also presented.

Under the assumption of Gaussian profile, the beam radius at any point along the *z* direction can be calculated as follows:^26^

(4) $$w\left(z\right)=\omega_{0}\left[\sqrt{1+{\left(\frac{z}{z_{R}}\right)}^{2}}\right],$$

where $z_{R}$ is the Rayleigh range defined as ,

(5) $$z_{R}=\frac{\pi w_{0}2}{\lambda }.$$

The expansion of the beam from the beam waist point is defined as divergence angle and is given by Equation (6):^26^

(6) $$\theta =\frac{2\lambda }{\pi w_{0}},$$

where *λ* is the wavelength of the beam.

The shape of the Gaussian beam is described by the beam waist *w*_0_ where the beam diameter becomes minimum.

**FIG. 5.**
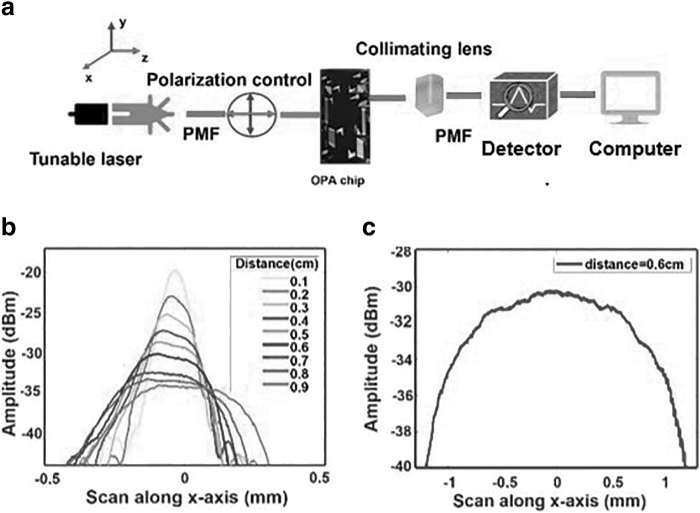
**(a)** Sketch of the experimental characterization setup. **(b)** Measured horizontal intensity distributions at different distances. **(c)** Measured vertical intensity distribution.

The sketch of the experimental setup used to measure the power spectra and spot sizes from the lenses is described in Figure 5. A semiconductor tunable laser (Santec TSL-510) source is coupled to the waveguide chip with a polarization maintaining fiber. The wavelength is 1.5 μm and the intensity is 13 dBm. The input and the output micrometer stages can be moved in all three directions, that is, *x*, *y*, and *z*, whereas the middle stage containing the chip can only be moved in *x* direction. A multimode fiber is collecting the output light emerging from the chip and is connected to the detector (Hewlett Packard 81635A). The intensity can be observed on a computer screen.

The output of the first-generation OPA chip without a lens is scanned horizontally (*y* direction), as shown in Figure 5b. The beam continuously diverges, and the beam waist clearly lies at the edge of the chip, as expected. The output line is properly confined in the horizontal direction and offers a very narrow width. The chip is offering the horizontal collimation and the collimation lens is just needed for vertical direction.

A vertical scan of the output results in the profile shown in Figure 5c. Repetitive measurements at different distances from the chip shows that the light is not collimated vertically. The scan along the height of the output beam came out to be 2.3 mm at distance 0.6 cm away from chip. Thus, there is a need of collimating lens in the vertical direction. In this article, we are presenting the characterization result of two collimating lenses, that is, a commercially available straight cylindrical lens (LJ1598L2-C) and a new design of 3D printed curved cylindrical lens. The focal length of LJ1598-C is 3.9 mm, whereas the height of the lens is 4 mm.

**FIG. 6.**
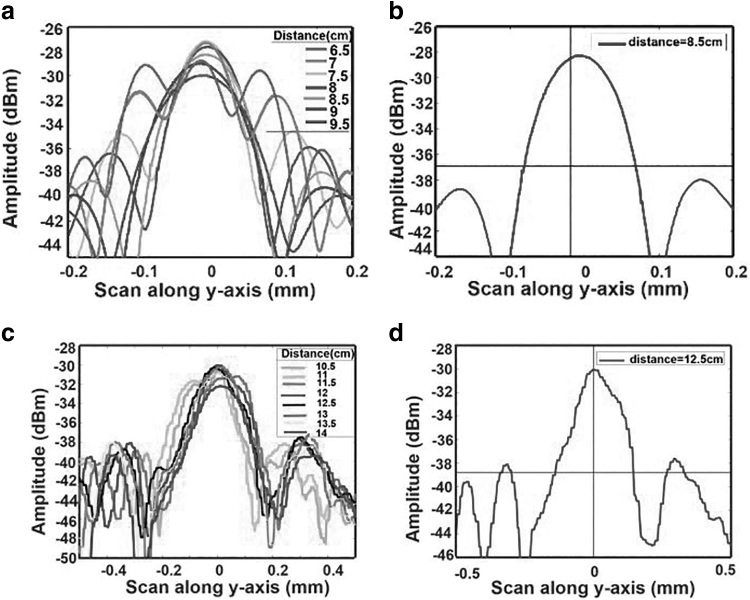
**(a)** Measured vertical intensity distributions at different distances along *z* axis (a) Lens LJ1598L2-C. **(b)** Measured intensity distribution at beam waist of LJ1598L2-C. **(c)** 3D printed lens. **(d)** Measured intensity distribution at beam waist of 3D printed lens.

The intensity distributions are measured for the lens LJ1598L2-C at different distances from the chip in the vertical direction, as shown in Figure 6a. The beam width increases before and after the waist point, that is, 8.5 cm in Figure 6b.

**FIG. 7.**
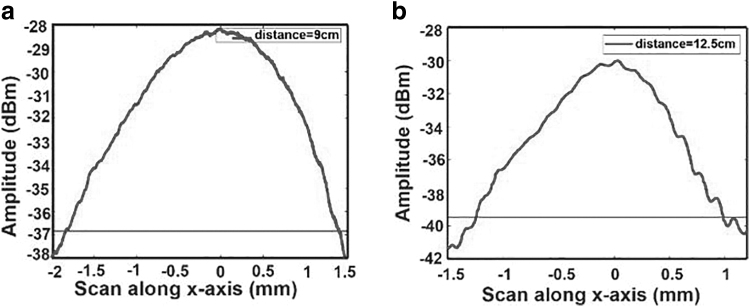
Horizontal spot size of the beam at the location of beam waist. **(a)** LJ1598-C lens. **(b)** 3D printed lens.

The calculated beam waist (*w*_0_) from the experiment performed in the laboratory is ∼0.07 mm, as seen from Figure 7b. From Equations (2) and (4), we can easily calculate the Rayleigh range and divergence angle to be 9.93 mm and 7.04 mrad (0.403°), respectively. Side lobes can be observed apart from the main peaks because of spherical aberration. It can be corrected using the aspheric or aplanatic lenses. However, measurements are not much affected by it in our particular case.^33^

The beam is further scanned along the horizontal direction, see Figure 7a. The horizontal spot size is measured to be 3.23 mm. The narrow 37 channel waveguides array limits the collimation distance of the whole laser beam, because the horizontal spot size of 3.23 mm is much larger than the vertical spot size of 0.07 mm at beam waist position.

The commercial lenses offer good quality beam collimation in the straight direction. However, they are not suitable for beam steering at large angles. Thus, we are proposing the dedicated 3D printed curved lenses (see Fig. 4c), which can offer beam steering at wide angles. The 3D printed lens is scanned along the vertical direction and the result is presented in Figure 7c. These measurements yield a beam waist $w_{0}≃0.14mm$ , a Rayleigh range $z_{R}≃40.18mm$, and a divergence θ≃3.5mrad (0.20°). The Rayleigh range of the 3D printed lens is about four times longer and divergence angle about twice smaller than the commercial LJ1598L2-C lens. Moreover, one can observe a drastic reduction of side lobes with the 3D printed lens.

The horizontal intensity scan is shown in Figure 7b. The horizontal spot size with 3D printed lens is 2.22 mm, which is comparable with the measurement done with the commercial lens, that is, 2.87 mm.

**FIG. 8.**
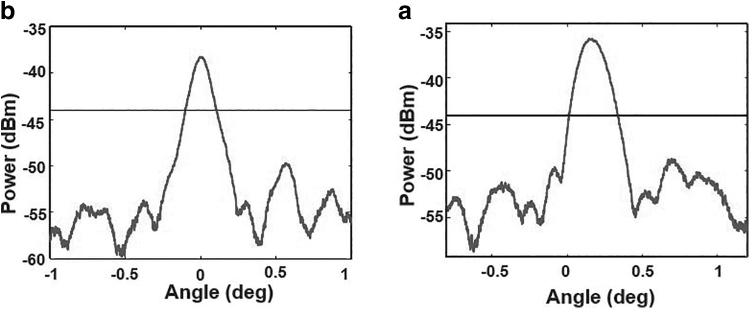
Measured vertical spot size of the beam 48 cm away from the chip. **(a)** Commercial LJ-1598 lens. **(b)** 3D printed lens.

The second OPA generation with 512 output channels has been characterized at 48 cm away from the chip. The vertical beam divergence after the commercial lens can be seen in Figure 8a and compared with the 3D printed lens (Fig. 8b). The beam divergence value with the commercial lens is 0.2° and with 3D printed lens is 0.319°, which is greater this time.

Although the 3D printed lens shows a clear improvement compared to the commercial lens, it is to be remarked that glass-based lenses remain better than 3D printed lenses in terms of surface roughness. The 3D printed lens has spherical aberration correction because of the conical surface. The shape is better than in commercial glass spherical surface lenses, which might give better values in short range measurements. A correction of spherical aberrations of the commercial lens would give better results. However, for large angles, laser beam steering gives better results when the 3D printed curved cylindrical lens is used, because the shape of straight lens changes as a function of the angle.

## Conclusion

In this article, we have compared commercial lenses and 3D printed lenses on two generations of OPA. The first generation of OPA contains a waveguide array of 108-μm wide, which is narrow for long-range collimation. The second generation of OPA contains 512 waveguides for a total width of the array larger than 1 mm. The maximum beam steering angle for both 37 and 512 channel OPA is nearly identical, that is, 30°, but the beam movement is 0.35°/nm and 0.6°/nm, respectively.

The collimation distance increased by at least an order of magnitude with wider waveguide array. We have demonstrated a higher performance with the 3D printed lens, although this first version is still comparable to the commercial collimating lenses, mainly due to aberration coming from the surface roughness. However, it is possible to develop machine curved cylindrical lenses from existing glass- or polymer-based materials, but it is time-consuming and much more expensive. 3D printing of lenses offers faster and cost-effective way to develop optimal lens design for the curved cylindrical lens at the prototyping phase before large-scale manufacturing. A reduced surface roughness would improve the results obtained with the 3D printed lens in long-range applications.
